# Interferon-Free Hepatitis C Treatment before and after Liver Transplantation: The Role of HCV Drug Resistance

**DOI:** 10.3390/v7092864

**Published:** 2015-09-23

**Authors:** Bruno Roche, Audrey Coilly, Anne-Marie Roque-Afonso, Didier Samuel

**Affiliations:** 1 AP-HP Hôpital Paul-Brousse, Centre Hépato-Biliaire, 12-14 avenue Paul Vaillant-Couturier, Villejuif, F-94800, France; bruno.roche@aphp.fr (B.R.), audrey.coilly@aphp.fr (A.C.); 2 Univ. Paris-Sud, UMR-S 1193, Université Paris-Saclay, 12-14 avenue Paul Vaillant-Couturier, Villejuif, F-94800, France; 3 Inserm, UMR-S 1193, Université Paris-Saclay, Villejuif F-94800, France; 4 Hepatinov, Villejuif, F-94800, France; 5 AP-HP Hôpital Paul-Brousse, Laboratoire de Virologie, Villejuif F-94800, France

**Keywords:** liver transplantation, hepatitis C, antiviral therapy, direct-acting antiviral, interferon, ribavirin, boceprevir, telaprevir, sofosbuvir, simeprevir, daclatasvir, ledipasvir, paritaprevir, ombitasvir, dasabuvir

## Abstract

Hepatitis C virus (HCV) infection is one of the leading causes of end-stage liver disease and the main indication for liver transplantation (LT) in most countries. All patients who undergo LT with detectable serum HCV RNA experience graft reinfection progressing to cirrhosis within five years in 20% to 30% of them. Obtaining a sustained virological response (SVR) greatly improves overall and graft survival. Until 2011, standard antiviral therapy using PEGylated interferon (PEG-IFN) and ribavirin (RBV) was the only effective therapy, with an SVR rate around 30% in this setting. For patients infected with genotype 1, first generation NS3/4A protease inhibitors (PIs), boceprevir (BOC) or telaprevir (TVR), associated with PEG-IFN and RBV for 48 weeks have increased the SVR rates to 60% in non-transplant patients. However, tolerability and drug-drug interactions with calcineurin inhibitors (CNI) are both limiting factors of their use in the liver transplant setting. Over recent years, the efficacy of antiviral C therapy has improved dramatically using new direct-acting antiviral (DAA) agents without PEG-IFN and/or RBV, leading to SVR rates over 90% in non-transplant patients. Results available for transplant patients showed a better efficacy and tolerability and less drug-drug interactions than with first wave PIs. However, some infrequent cases of viral resistance have been reported using PIs or NS5A inhibitors pre- or post-LT that can lead to difficulties in the management of these patients.

## 1. Introduction

Despite the recent improvements in treatment strategies, hepatitis C virus (HCV) infection remains one of the leading causes of end-stage liver disease and a main indication for liver transplantation (LT) [1]. All patients who undergo LT with active HCV infection experience graft reinfection. The natural history of HCV graft infection is accelerated with around 30% progressing to cirrhosis within five years. Recurrent HCV infection is the most frequent cause of death and graft loss in these patients [2,3]. Survival reaches 61%–75% and 68% at five and 10 years post-LT, respectively [4,5,6]. The outcome of transplant patients with cirrhosis on the graft is severe, with a rate of decompensation of around 40% at one year [2]. Two to 8% of patients experience severe HCV recurrence, known as fibrosing cholestatic hepatitis (FCH), characterized by high HCV RNA levels and a very poor prognosis in patients who do not respond to antiviral therapy [7]. Certain factors are associated with an accelerated progression of fibrosis in patients with recurrent HCV infection [7]. For example, high HCV RNA levels in both serum and the liver at the time of or early post-LT [8,9], female donor, older donor age, black recipient race, steatosis of the graft, the degree of human leukocyte antigen (HLA) matching or the IL28B genotype of the donor and the recipient and HIV co-infection [3,10,11] are associated with an increased rate of fibrosis progression on the graft. The management of recurrent HCV infection is directly related to the immunosuppressive regimen of the recipient for two reasons. First, the degree and composition of the immunosuppressive regimen influence the progression of fibrosis [12]. Second, potential drug-drug interactions must be taken into account before using antiviral therapy, mainly protease inhibitors (PIs) [13]. Obtaining a sustained virological response (SVR) can greatly improve overall and graft survival [14,15,16]. Until 2011, dual therapy based on the combination of PEGylated interferon (PEG-IFN) and ribavirin (RBV) was the only effective therapy leading to an SVR rate of 20% to 30% in genotype 1 patients and 40% to 50% in genotype 2 and 3 patients [15]. In non-transplant patients infected with genotype 1, the first generation NS3/4A protease inhibitors (PI) boceprevir (BOC) or telaprevir (TVR) associated with PEG-IFN and RBV increased the SVR rates to 50% to 70% [17,18,19,20]. However, this advance is limited in transplant recipients related to poor tolerability and drug-drug interactions with calcineurin inhibitors (CNI) [17,18,19,20]. In the non-transplant setting, using new direct-acting antiviral (DAA) agents, the efficacy of antiviral C therapy has improved dramatically over recent years leading to SVR rates over 90%, without PEG-IFN and/or RBV [21,22,23,24,25]. Results available for transplant patients confirmed a better efficacy and tolerability and less drug-drug interactions than with first wave PIs [26,27,28,29,30]. Some infrequent cases of occurrence of resistant-associated variants (RAVs) have been reported using PIs or NS5A inhibitors [31]. The occurrence of viral mutations before or after LT could lead to difficulties in the management of these patients. This review describes the management of HCV infection pre- and post-LT and the impact of viral mutations during antiviral therapy.

## 2. New Direct-Acting Antiviral Agents

Several classes of DAAs have reached the market and target different viral non-structural proteins, including the NS3/4A protease, the NS5B polymerase and the NS5A protein [32]. Their efficacy and barrier to resistance may depend on the HCV genotype/subtype [33].

The second wave of first generation NS3/4A PIs includes simeprevir (SIM) and ritonavir-boosted paritaprevir (paritaprevir/r). These drugs are active against all genotypes, except genotype 3, due to the natural polymorphism D168Q that confers resistance to all available PIs. Reduced efficacy was also shown for patients infected with HCV genotype 1a harboring a Q80K polymorphism [34]. They have a high potency, a low genetic barrier to resistance and share extensive cross-resistance. Resistance-associated variants (RAVs) emerging after treatment failure have been shown to be short-lived with restoration to the wild-type within 1 to 2 years [35].

Nucleos(t)ide analog NS5B polymerase inhibitors, such as sofosbuvir (SOF), are active against all genotypes and have a high potency and a high barrier to resistance. Indeed, the S282T mutation conferring resistance to this class dramatically impairs viral replication and has been rarely detected in patients failing SOF-based treatments. Non-nucleoside NS5B polymerase inhibitors (NNIs), such as dasabuvir, interact with the viral polymerase outside the catalytic site and prevent conformational changes critical to its function. Different allosteric binding sites determine specific resistance patterns, thus limited cross-resistance exists between distinct NNI classes. These compounds are active against genotype 1, but display higher efficacy to 1b than 1a. They have a low potency and the lowest barrier to resistance among DAAs.

NS5A inhibitors, such as daclatasvir (DCV), ledipasvir (LDV) and ombitasvir, are active against all genotypes and have a high potency and a low barrier to resistance. Higher rates of SVR to DCV-containing regimens have been observed for patients infected with HCV genotype 1b, compared to 1a, explained by subtype differences in barriers to resistance. Mutations at positions 31 and/or 93 confer a broad cross-resistance to NS5A inhibitors. Unlike NS3 RAVs, NS5A RAVs selected during treatment are relatively fit in terms of replication capacity and might persist for a long period of time after treatment discontinuation [31,36].

Combination of DAAs, which target different steps of viral replication, should provide additive or synergistic antiviral potency and prevent the emergence of DAAs resistance [32]. Two all-oral, IFN-free strategies are being investigated: (1) SOF-based strategies use SOF as the backbone of therapy, in combination with RBV (SOF + RBV) or with one or two DAAs, with or without RBV (SOF + LDV or SIM or DCV); (2) SOF-free triple combination strategies combine drugs with a low barrier to resistance: PIs, NS5A inhibitors and non-NUC NS5B inhibitors (paritaprevir/r + ombitasvir + dasabuvir).

## 3. Pre-Transplant Antiviral Treatment

Before the availability of interferon-free regimens, the best strategy to prevent recurrent infection was to eradicate HCV prior to LT. Indeed, SVR rates in transplant patients were poor, and patient’s management was made more complex by recurrent HCV. However, HCV treatment in patients awaiting LT for end-stage liver disease was also challenging. Interferon-free regimens provide shorter, safer and more efficient therapy and may dramatically simplify patient’s management. Using new DAAs, it is expected that HCV eradication before LT prevents HCV recurrence, but also will rescue some patients from the need for LT, as observed with antiviral therapies in decompensated HBV infection [21,22,23,24,25]. The first option is to achieve SVR before LT; in this case, there is no recurrence of HCV infection on the graft. The second option is to achieve on treatment undetectable HCV RNA at LT. In a recent study, aimed to prevent HCV recurrence on the graft, SOF plus RBV was used in 61 LT candidates (genotype 1: 74%, previously treated: 75%, median Model for End-Stage Liver Disease (MELD) score = 8; 6 to 14) listed for compensated cirrhosis with hepatocellular carcinoma, until the time of LT or for up to 48 weeks [37]. Fifteen patients discontinued treatment before LT, in nine cases for virologic failure; thus, 46 patients underwent LT and were analyzed for HCV recurrence rates. Of these, 43 had undetectable HCV RNA at the time of LT, and 30 (70%) still had undetectable HCV RNA at 12 weeks after LT. The strongest predictor of post-LT SVR was the number of consecutive days with undetectable HCV RNA before LT. Patients with more than 30 days of HCV RNA undetectability had a 95% chance of no HCV recurrence after LT. Safety analysis was good. Resistance analysis was performed in 29 patients who had virologic failure or relapse after LT, without evidence of selection of RAVs to SOF.

However, there are some limitations in pre-LT antiviral therapy: the use of antiviral drugs, mainly SOF, is limited in patients with severely impaired kidney function; more data are needed to understand the consequences of virologic failure following DAA therapy, as well as the development of effective strategies to treat these patients pre- or post-LT; and lastly, the duration of HCV therapy before LT is unpredictable. It is very relevant to choose the most effective antiviral combination to minimize the possibility of virological relapse and the selection of RAVs, because they could infect the graft and persist for a prolonged time in the setting of immunosuppression and might hamper antiviral therapy in the case of severe hepatitis C recurrence. Indeed, as said above, wild-type virus has been shown to rapidly outgrow less fit NS3 RAVs variants [35], but unlike NS3 RAVs, NS5A RAVs might persist for a long period of time after treatment discontinuation [31,36,38]. Regarding retreatment results, there is no reason to contraindicate to LT patients with HCV drug resistance, especially if resistance relates to a single class of DAAs, because DAAs targeting other viral proteins remain fully efficient. For example, for patients who failed on combination therapy including first generation PI, 24 weeks of SOF/DCV yielded SVR rates of 95% and 100% without and with RBV, respectively [39]. Similar results have been obtained using LDV in the ION-2 and SIRIUS studies [24,40]. Currently, there are few data concerning retreatment after the combination of DAA failure, and the clinical relevance of resistance to HCV DAAs, owing to preexisting polymorphisms or selection after exposure to an antiviral drug, remains unclear. Indeed, in phase 3 trials using SOF + LDV, up to 18% of genotype 1-infected naive or PI treatment-experienced patients have NS5A polymorphisms at baseline with SVR rates ranging from 89% to 96% [23,24,41]. Interestingly, no relapse occurred in subjects with baseline NS5A RAVs in the LDV/SOF 24 weeks and LDV/SOF + RBV 12 weeks, suggesting that extending treatment duration or adding RBV may optimize response rates in this subset of patients. Actually, in treatment-experienced patients with cirrhosis treated with LDV/SOF 24 weeks or LDV/SOF + RBV 12 weeks, baseline NS5A polymorphisms were present in 16% of patients, and though baseline NS5A RAVs seemed overrepresented, there was no significant difference in SVR rates based on baseline polymorphisms: 92% SVR *vs.* 98% SVR in patients without RAVs [40]. Post-transplant outcome of patients with multi-resistant viruses to PI and NS5A inhibitors is questionable. In the absence of new therapeutic classes, these patients may be unmanageable, leading to systematic HCV recurrence and, thus, poorer prognosis of graft and patient survival. The decision of retreatment before LT has to take into account genotype/subtype, resistance profiles, use of ribavirin and treatment duration [42]. The possibility to obtain an undetectable viral load during treatment in the pre-LT period (*i.e.*, on treatment virological response) should be evaluated.

At the present time, no data are available on the efficacy and safety of new DAA regimens in decompensated cirrhosis (*i.e.*, MELD score > 20). In this group of patients, the pharmacokinetics of DAAs is modified. There is also a risk of liver decompensation during antiviral therapy or after viral breakthrough. Consequently, for patients with decompensated cirrhosis, candidates for LT, antiviral therapy could be delayed after LT. The management of patients with less than one month of viral undetectability during antiviral therapy before LT is unknown. Results concerning the continuation of antiviral therapy immediately after LT are not yet reported and should be evaluated.

## 4. Management of Recurrent HCV Infection

Prophylactic post-transplant antiviral therapy aiming at avoiding/limiting graft reinfection using anti-HCV monoclonal antibodies post-LT is still in evaluation [43]. Another strategy may be applied in the future using entry inhibitors in the immediate post-transplant period, since entry inhibitors have been shown to successfully prevent liver graft infection in animal models [44,45]. Pre-emptive treatment after transplantation and before the occurrence of hepatitis on the graft within one month post-LT gave low SVR due to a poor tolerance and a high rate of treatment discontinuation using IFN-based therapy [46,47,48,49,50]. This strategy needs now to be re-evaluated with an IFN-free antiviral regimen.

It is generally accepted that antiviral therapy after LT should be initiated in the presence of histologically-proven HCV recurrence. However, this decision must also take into account age, the patient’s general condition, previous therapy failures, anemia, renal insufficiency, immunosuppression, drug-drug interactions and the stage of fibrosis, usually >F1 on the METAVIR score (algorithm for histological evaluation of hepatitis C). Antiviral therapy should be initiated in the presence of severe fibrosis and rapid progression of fibrosis with a higher risk of graft loss, especially FCH. If a liver graft biopsy is not performed, other non-invasive markers can help to make the treatment decision. A cut-off value of 8.7 kPa for liver stiffness had a sensitivity and a negative predictive value for significant fibrosis and portal hypertension >0.90 in all cases [51]. The measurement of the hepatic venous pressure gradient can help, with a gradient >6 mmHg for significant fibrosis [52]. Although non-invasive markers can discriminate the stage of fibrosis, regular protocol biopsies of the graft are essential before antiviral therapy is begun to obtain crucial data, such as the progression of graft fibrosis, the presence of rejection, biliary obstruction or the degree of steatosis. Finally, we have reported that tolerance to IFN-based therapy decreases significantly in patients with fibrosis stage ≥3, suggesting that antiviral therapy should be initiated before advanced fibrosis develops [53].

There is no doubt that the best timing to treat HCV recurrence may be updated in the very near future. The current strategy to wait for significant fibrosis on the liver graft before initiating antiviral treatment is supported by the poor tolerability of IFN-based regimens early after LT. The next generation of IFN-free DAAs should result in earlier treatment, although there is currently no firm data to support this.

## 5. Post-LT Treatment with IFN-Based Regimens

Several studies using PEG-IFN/RBV have shown SVR rates of 18% to 45%, and three systematic reviews showed SVR rates of 30% (20% to 30% in genotype 1 patients and 40% to 50% in genotype 2 and 3 patients) [15,54,55]. Factors associated with SVR are non-genotype 1, the absence of prior antiviral therapy, an early virological response (EVR) or rapid virological response (RVR), adherence to therapy, low pretreatment viral load, low fibrosis stage and a favorable donor and/or recipient IL28B genotype [6,10,15,54,55,56]. Tolerance to interferon-based therapy is a major issue. Dose reductions of RBV and/or PEG-IFN were necessary in around 70% of patients, and the rate of treatment discontinuation was approximately 30% [15,54,55]. Several studies have been published on the results of BOC and TVR use after LT. We performed a multicenter study of 37 patients treated with triple therapy (TVR *n* = 19, BOC *n* = 18) after LT [18]. Finally, a SVR 12 was obtained in one of the five eligible patients (20%) in the TVR group and five of the seven eligible patients (71%) in the BOC group. Six patients (16%) developed viral breakthrough. Complete NS3 sequence information was obtained in seven patients who experienced treatment failure, non-response or a breakthrough. At least one mutation related to PI was detected in all of them.

Burton *et al.* reported a retrospective cohort of transplant recipients with recurrent genotype 1 infection treated with either BOC- (*n* = 8) or TVR- (*n* = 73) based triple therapy at six U.S. transplant centers [17]. The intent-to-treat SVR 12 rate was 63% (51/81). Unfortunately, no RAVs analysis was performed, either at baseline or at the time of the virological relapse. A phase 3b study of the use of TVR (REPLACE) in 74 stable, non-cirrhotic, treatment-naive post-LT patients showed an SVR 12 rate of 67% [19].

Overall, first generation PI-based PEG-IFN-containing antiviral therapy achieved an approximately two-fold higher SVR rate than has historically been achieved with PEG-IFN and RBV. However, therapy is associated with significant hematological toxicity, an increased risk of sepsis, drug-drug interactions and, ultimately, mortality. This antiviral regimen is no longer recommended when IFN-free regimens are available.

## 6. Post-LT Treatment with IFN-Free Regimens

Although data are currently scarce, it is anticipated that IFN-free regimens will be highly effective and safe in the post-LT setting (Table 1) [26,27,28,29,30,57,58,59].

**Table 1:** Results of IFN-free regimens in liver transplant recipients with hepatitis C recurrence. DAA: direct-acting antiviral; LT: liver transplantation; SVR: sustained virologic response; SOF: sofosbuvir; RBV: ribavirin; IFN: interferon; PI: protease inhibitor; NA: not available; SIM: simeprevir; DCV: daclatasvir; LDV: ledipasvir; FCH: fibrosing cholestatic hepatitis.

DAA Regimen (Reference)	Patients	Time Since LT Median (Range)	Genotype	Failure to Previous Therapy	Fibrosis Stage	SVR 12	Virologic Failure
**SOF-RBV 24 Weeks [28]**	40	4.3 years (1.0 to 10.6)	G1a: 22 (55%)	IFN ± RBV: 25 (71%)	≤F2: 15 (37%)	70%	Relapse
			G1b: 11 (28%)	PI: 9 (26%)	>F2: 25 (63%)		
**SOF-RBV (80 patients) SOF-RBV-PEG-IFN (24 patients) 24–48 Weeks [26]**	Early recurrence 52	8.4 months (4.8 to 12.7)	G1a: 22 (42%)	NA	NA	73%	Relapse
			G1b: 23 (44%)				
	Cirrhosis 52	53.1 months (33.1 to 92.1)	G1a: 14 (27%)	NA	F4	43%	Relapse
			G1b: 26 (50%)				
**Paritaprevir/r-ombitasvir-dasabuvir-RBV 24 weeks [27]**	34	39.5 months (12.9 to 136.4)	G1a: 29 (85%)	Naive post-transplant	≤F2	97%	RAV in NS3, NS5A and NS5B (1 patient)
			G1b: 5 (15%)				
**SIM-SOF ± RBV 12 weeks [30]**	123	32 months (2 to 317)	G1a: 74 (60%)	IFN ± RBV: 85 (69%)	≤F2: 85 (70%)	90%	NA
			G1b: 43 (35%)	PI: 15 (12%)	>F2: 37 (30%)		
**LDV-SOF-RBV 12 to 24 weeks [29]**	162	NA	G1a: 114 (70%)	IFN ± RBV: 98 (60%)	F0 to F3 or F4 Child–Pugh A	96% (12 w)	NS5A variants were present in 85% of patients who relapse (6 patients)
			G1b: 47 (29%)	PI: 21 (13%)		98% (24 w)	
	52	NA	G1a: 38 (73%)	IFN ± RBV: 29 (56%)	F4 Child–Pugh B	85% (12 w)	
			G1b: 13 (25%)	PI: 12 (23%)		88% (24 w)	
	9	NA	G1a: 7 (78%)	IFN ± RBV: 7 (78%)	F4 Child–Pugh C	60% (12 w)	
			G1b: 2 (22%)	PI: 1 (11%)		75% (24 w)	
	6	NA	G1a: 5 (83%)	IFN ± RBV: 5 (83%)	FCH	100% (12w)	
			G1b: 1 (17%)			100% (24 w)	
**DCV-SOF-RBV 12 weeks [58]**	53	>3 months	G1a: 31 (58%)	58%	≤F2: 23 (43%)	94%	NS5A variants were present in all patients who relapse (3 patients)
			G1b: 10 (19%)		>F2: 29 (55%)		
			G3: 11 (21%)				
**DCV-SOF DCV-SOF-RBV 12 to 24 weeks [59]**	14	86.1 ± 77.5	G1: 92%	42.7%	>F2: 56%	97%	Among the 2 patients with virologic failure, NS5A variants were present in the only tested patient
	116	62.6 **±** 56.9	G1: 80%	54.5%	>F2: 49%	96%	

In a phase 2 prospective, multicenter, open-label pilot study, 40 LT recipients with compensated recurrent hepatitis C (cirrhosis: 40%, genotype 1: 83%, previously treated: 88%, previous first generation PI failure: 23%) were treated with SOF + low ascending-dose RBV for 24 weeks [28]. All patients achieved RVR and end of treatment (EOT) virologic response, and 70% achieved SVR 12 (75% in patients without cirrhosis and 62.5% in patients with cirrhosis). Relapse accounted for all cases of viral failure. No case of resistance was reported, and the therapy was safe and well tolerated, with no deaths, graft losses, rejection episodes or drug-drug interactions with immunosuppressive drugs. Relapse rates were not influenced by RBV dose or exposure. The results of a compassionate access program of SOF and RBV ± PEG-IFN (*n* = 24) up to 48 weeks for 104 LT recipients with severe HCV recurrence were: early severe recurrence <12 months from LT, including 10 cases of FCH (*n* = 52), or compensated or decompensated graft cirrhosis >12 months from LT (*n* = 52) [26]. Patients have a life expectancy without antiviral therapy of less than 12 months, and the median MELD was 15 (6 to 43). The SVR 12 rate excluding patients who underwent re-transplantation (*n* = 12) was overall 59%, 73% for patients with early severe recurrence and 43% for patients with cirrhosis. Of the 103 patients with available data, 59 (57%) were classified as having an improvement of clinical condition; 23 (22%) had unchanged clinical status; and 21 (21%) had worsened clinical status or had died. Overall median MELD scores decreased from 16 to 8. Thirteen patients died during the study from liver failure, reflecting the severity of liver disease at study entry.

The SOLAR-1 study assessed the efficacy and safety of LDV, SOF and RBV during 12 or 24 weeks for transplanted patients infected by genotype 1 or 4 without cirrhosis (*n* = 111), with Child–Pugh A cirrhosis (*n* = 51), Child–Pugh B cirrhosis (*n* = 52), Child−Pugh C cirrhosis (*n* = 9) or FCH (*n* = 6) [29]. SVR 12 was achieved by 96% and 98% of patients without cirrhosis or Child–Pugh A cirrhosis, by 85% and 88% of patients with Child–Pugh B cirrhosis, by 60% and 75% of patients with Child–Pugh C cirrhosis and by all patients with FCH receiving 12 or 24 weeks of therapy, respectively. Twelve weeks of therapy were as effective as 24 weeks. At baseline, 14% of patients had NS5A RAVs that conferred reduced susceptibility to LDV. Relapse occurred in 7% of patients with baseline RAVs as compared to 4% in patients without baseline RAVs. No relapses were observed for patients who received 24 weeks of therapy. At the time of virological failure, among patients who relapsed, 85% were observed to have NS5A variants. No resistant variants to SOF were observed. The SOLAR-2 study, following the same design, reports the same results. SVR 12 was achieved by 95% and 98% of patients without cirrhosis or Child–Pugh A cirrhosis and by 85% and 88% of patients with Child–Pugh B-C cirrhosis receiving 12 or 24 weeks of therapy, respectively [57].

A multicenter U.S. study reports on the efficacy, safety and tolerability of SIM and SOF with (80%) or without (20%) RBV for 12 weeks in 123 liver transplant recipients infected by genotype 1 [30]. The EOT response rate by intention to treat analysis was 97% (119/123 patients). Two patients discontinued treatment prematurely due to serious adverse events one patient developed viral rebound between weeks 4 and 6; and one patient developed viral breakthrough at week 9. The SVR 12 response rate was 90% (94/105 patients). Eight patients developed virological relapse within four weeks after treatment completion. Unfortunately, no RAV analysis was performed, either at baseline or at the time of the virological relapse. Treatment was well tolerated, except one death, possibly due to drug-related lung injury.

Results concerning the use of DCV and SOF regimen were reported as the 2015 European association for the study of the liver (EASL) meeting. In the ALLY-1 study, 53 transplanted patients (cirrhosis: 30%; genotype 1: 77%, previously treated: 58%) were treated with DCV, SOF and RBV for 12 weeks. An SVR 12 was observed in 50 patients (overall 94%; genotype 1: 94%; genotype 3: 91%). Among three patients who relapsed, all were observed to have NS5A variants [58]. In the French prospective CUPILT study, 130 transplanted patients (cirrhosis: 31%; genotype 1: 82%; previously treated: 48%) were treated with DCV, SOF with or without RBV for 12 (*n* = 14) or 24 weeks (*n* = 116) [59]. An SVR 12 was observed in 67% and 100% of patients with and without RBV in the 12-week arm and 96% and 97% of patients with and without RBV in the 24-week arm. Among the two patients with virologic failure, NS5A variants were present in the only tested patient. RBV does not seem mandatory. The tolerance profile was good; however, attention should be paid to renal function, as a significant decrease has been observed.

In another phase 2 study, 34 LT recipients with mild recurrent hepatitis C (genotype 1, fibrosis ≤2, treatment-naive post-LT and ≥12 months post-LT) were treated with a quadritherapy, including paritaprevir/r, dasabuvir, ombitasvir and RBV, for 24 weeks [27]. Adjustment of CNI dose will be required because of the inhibition of Cytochrome (CYP)-3A4 by the ritonavir-boosted PI paritaprevir (seven-fold increase in Tacrolimus half-life, three-fold increase in Cyclosporine half-life) [60]. All patients achieved RVR and EOT viral response, and 33 of 34 (97%) achieved SVR 12. One patient had a relapse. This patient had RAVs in NS3, NS5A and NS5B at the time of relapse, none of which were present at baseline. The regimen was well tolerated without death, graft loss or rejection episode.

Based on the results of these studies, HCV management guidelines for pre- and post-transplant patients were reported by the American Association for the Study of Liver Diseases (AASLD), as well as the Infectious Diseases Society of America (IDSA) and EASL [42,61]. For patients with genotype 1 or 4 infection, SOF + SIM with RBV for 12 weeks is recommended. The alternative regimen for this group is SOF + LDV or DCV with RBV (12 weeks) or without RBV (24 weeks) or paritaprevir/r, dasabuvir (for genotype 4), ombitasvir and RBV for 12 to 24 weeks. For patients with genotype 2 infection, SOF + RBV 12 to 24 weeks is recommended. The alternative regimen for this group is SOF + DCV with RBV 12 weeks. For patients with Genotype 3 infection, SOF + RBV 24 weeks or SOF + DCV with RBV 12 weeks is recommended.

In conclusion, it is important to maximize the treatment in that specific setting. The aim must be to use the most effective treatment that provides the highest SVR rate, avoiding the risk of occurrence of RAVs. IFN-free regimens appear to be highly effective (80% to 90%) in LT recipients, even in patients with FCH [62]. Actually, very few data are available on the efficacy and safety of new DAA regimens in patients with decompensated cirrhosis (*i.e.*, MELD score >20) post-LT [26]. More studies are needed for this group of patients with advanced disease. Safety profiles are similar and favorable amongst all IFN-free regimens. However, some limitations should be highlighted.

## 7. Unmet Medical Needs Regarding DAA in Transplant Recipients

Drug-drug interaction is still a main issue in our field; besides, they are less potent using second generation DAAs compared to first generation PIs. Except SOF, second generation PIs and NS5A inhibitors are substrates and inhibitors of the CYP-3A4 and Pg-p metabolic pathways and, thus, could interact with several drugs, such as immunosuppressive drugs [63]. Variation of the pharmacokinetics of CNI with DAAs could be observed, but most of them seem to be clinically irrelevant (Table 2). No adjustment should be applied with SOF, DCV and SIM, but we must remain vigilant before the availability of “real-life” data. In the phase 2 study described above including paritaprevir/r, dasabuvir, ombitasvir and RBV, a decrease of CNI dose was required due to the inhibition of CYP-3A4 by paritaprevir/r (seven-fold increase in Tacrolimus half-life, three-fold increase in Cyclosporine half-life) [27]. However, four patients experienced Tacrolimus overdose associated with a transient increase in creatinine level. In this study, no rejection was reported.

Another major issue is to know the metabolism of treatments. Indeed, if a DAA overdose may cause toxicity, under dosing may lead to a suboptimal exposure and, thus, promote the emergence of resistant variants. The pharmacokinetics of DAA depend on the selected drug, the presence of liver failure, renal failure and/or the concomitant prescription of other drugs that can interact (Table 3) [64,65,66,67,68]. Usually, DAAs have a liver metabolism. The use of PIs in Child-Pugh C patients is currently not recommended. SOF has no hepatic metabolism, but a renal one. SOF is not recommended in patients with creatinine clearance below 30 mL/min until the appropriate dosage is determined. A phase 2b, open-label study of 200 mg or 400 mg SOF + RBV for 24 weeks in genotype 1 or 3 HCV-infected subjects with renal insufficiency is ongoing (Clinicaltrials.gov:NCT01958281). Its use associated with LDV in kidney transplant patients is under investigation (Clinicaltrials.gov:NCT02251717).

Finally, non-adherence leads to suboptimal exposure to antiviral drug. It is associated with treatment failures and the emergence of RAVs, especially during the early phase of treatment. Adherence to treatment must be enhanced [69].

Actually, RBV is used in all IFN-based regimens and can decrease the safety of these treatments (Table 1). Anemia may limit or truncate ribavirin use in some patients. If these studies did not show superiority to a 12-week *versus* 24-week treatment duration, RBV seems mandatory to optimize the SVR rates and to avoid the emergence of RAVs.

**Table 2:** Potential drug-drug interactions between DAA and calcineurin inhibitors (CNI). http://www.hep-druginteractions.org/. 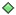
No clinically significant interaction expected; 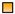 potential interaction that may require close monitoring, alteration of drug dosage or timing of administration; 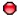 these drugs should not be co-administered; * cyclosporine use with simeprevir is not recommended, because cyclosporine increases the levels of simeprevir approximately 6-fold.

	Cyclosporine	Tacrolimus
Boceprevir	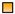	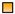
	Cyclosporine AUC increased +168%	Cyclosporine AUC increased +1016%
Telaprevir	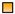	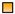
	Cyclosporine AUC increased by 4.64-fold	Tacrolimus AUC increased by 70.3-fold
Sofosbuvir	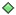	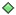
Sofosbuvir/ledipasvir	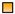	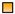
	Cyclosporine AUC decreased by 2%	Tacrolimus AUC increased by 13%
Daclatasvir	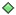	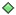
Simeprevir	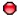	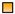
	Cyclosporine * AUC increased by 4.74-fold	Tacrolimus AUC increased by 79%
Ombitasvir, paritaprevir, ritonavir, dasabuvir	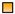	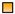
	Cyclosporine AUC increased by 5.82-fold	Tacrolimus AUC increased by 57.1-fold

**Table 3:** Pharmacokinetic changes according to liver and renal function. Clinically significant values are shown in bold.

Reference	Primary Metabolic Pathway	Hepatic Impairment			Avoid	Renal Impairment
		Mild	Moderate	Severe		
Sofosbuvir [64]	Renal	No modification	+1.26	+1.43		Not if CrCl <30 mL/min
Simeprevir [66]	Hepatic	No modification	**+2.44**	**+5.22**	Child C	Not if CrCl <15 mL/min
paritaprevir/r [67]	Hepatic	−0.71	**+1.62**	**+10.23**	Child C	Not studied in dialysis patients
Ledipasvir [65]	Hepatic	No modification	No modification	No modification		Not studied in dialysis patients
Daclatasvir [68]	Hepatic	−0.57	−0.62	−0.64		Not studied in dialysis patients
Ombitasvir [67]	Hepatic	+0.92	+0.70	+0.45		Not studied in dialysis patients
Dasabuvir [67]	Hepatic	+1.17	+0.84	**+4.19**	Child C	Not studied in dialysis patients
Ribavirin	Renal	No modification	No modification	No modification		Adjusted

Transplant recipients have high viral loads, making it easier to select for drug-resistant variants. Some cases of virologic failure have been reported related mainly to PIs or NS5A RAVs [26,27,29,58]. The choice of the IFN-free regimen should be determinate by previous antiviral treatment failure(s). Patients who failed after PEG-IFN/RBV combination pre- or post-LT do not respond differently to IFN-free regimens from treatment-naive patients. Patients infected with HCV genotype 1 who failed after a triple combination regimen of PEG-IFN/RBV and either BOC or TPV could have PIs RAV and should be treated preferentially with a SOF and NS5A regimen. Related to a high barrier to resistance, resistant HCV variants have been exceptionally reported with SOF, and they rapidly disappeared after treatment cessation. Thus, retreatment strategies should include SOF. In contrast, patients treated with a PI (SIM, paritaprevir/r) or an NS5A inhibitor (DCV, LDV, ombitasvir) who fail to achieve SVR select viruses with RAVs in the NS3 protease, NS5A, respectively, that confer drug resistance. Viruses resistant to PI decrease in proportion to become undetectable within a few months to two years after treatment cessation. In contrast, viruses resistant to NS5A inhibitors are fit and remain dominant for many years, after they have been selected. Currently, there are no data to firmly support retreatment recommendations for these patients. The use of HCV resistance testing prior to retreatment could be helpful to make a decision. Patients who failed on SOF alone or SOF plus RBV or SOF plus PEG-IFN/RBV can be retreated with a combination of SOF plus SIM (genotype 1 or 4), SOF plus DCV (all genotypes), or SOF plus LDV (genotypes 1, 4, 5 or 6), with paritaprevir/r, ombitasvir and dasabuvir (genotype 1), or with paritaprevir/r and ombitasvir (genotype 4). Patients who failed on a second wave DAA-containing regimen should be retreated with a drug with a high barrier to resistance (currently, SOF), plus one or two other drugs, ideally with no cross-resistance with the drugs already administered. Based on the results in difficult-to-treat patient populations, retreatment should be preferentially for 24 weeks and with ribavirin. Patients who failed on the triple combination of paritaprevir/r, ombitasvir and dasabuvir should be retreated with a SOF-based regimen. The value and safety of retreatment strategies combining three drugs, including SOF, a PI and an NS5A inhibitor, is unknown. Patients without an urgent need for treatment can wait until more data and/or alternative therapeutic options become available.

The rate of SVR seems lower in Child-Pugh B to C patients as compared to Child-Pugh A or non-cirrhotic patients [29].

## 8. Conclusions

Recurrent HCV is a constant, severe complication in liver transplant recipients and is the primary cause of graft loss and death in these patients. PEG-IFN/RBV has been extensively studied in this population. This regimen has limited efficacy in pre- and post-LT setting, and tolerance is poor. For patients infected with genotype 1, first generation PIs associated with PEG-IFN and RBV for 48 weeks have increased the SVR rates and allowed a shortened duration of therapy in most non-transplant patients. However, this advance is limited in transplant patients related to poor tolerability and drug-drug interactions with CNI. In contrast, new DAAs, mainly nucleoside/nucleotide analogue inhibitors, such as SOF and NS5A inhibitors, have a very high efficacy and lower toxicity and drug-drug interactions in the pre- and post-transplant setting and will dramatically change the face of LT for hepatitis C. The goal of antiviral therapy with new DAA regimens should be: first, viral eradication before LT to prevent graft re-infection and possibly, in some patients, rescue to LT (except for patients with hepatocellular carcinoma); second, viral eradication post-LT to improve long-term graft and patient survival and to reduce the need for re-LT. Some infrequent cases of viral mutations have been reported using PIs or NS5A inhibitors pre- or post-LT that can lead to difficulties in the management of these patients.
